# Parasitic Plant–Host Interactions: Molecular Mechanisms and Agricultural Resistance Strategies

**DOI:** 10.1002/advs.202519030

**Published:** 2026-02-12

**Authors:** Jiayang Shi, Qi Xie, Feifei Yu

**Affiliations:** ^1^ State Key Laboratory of North China Crop Improvement and Regulation College of Life Sciences Hebei Agricultural University Baoding China; ^2^ Institute of Genetics and Developmental Biology The Innovative Academy of Seed Design Chinese Academy of Sciences Beijing China; ^3^ National Center of Technology Innovation for Maize, State Key Laboratory of Crop Germplasm Innovation and Molecular Breeding Syngenta Group China Beijing China; ^4^ State Key Laboratory of Maize Bio‐breeding College of Grassland Science and Technology China Agricultural University Beijing China

**Keywords:** Cuscuta, Orobanche, parasitism, *Striga*, strigolactones (SLs)

## Abstract

Obligate parasitic plants, particularly members of the Orobanchaceae family, including *Striga* and *Orobanche*, greatly devastate crop production. Here, we synthesize recent advances in understanding the molecular and ecological dynamics underlying parasitic plant‐host interactions, focusing on critical stages of parasitism: germination, host detection, haustorium formation, and resource extraction. Orobanchaceous parasites exploit host‐derived strigolactones (SLs) to break seed dormancy, whereas *Cuscuta* species do not rely on SLs for germination. Instead, chemotropic responses to host‐exuded compounds and light signals guide the directional growth of their seedlings. Haustorium morphogenesis, initiated through host lignin‐derived quinones and redox‐sensitive compounds, establishes vascular connectivity enabling nutrient diversion. Meanwhile, host organisms employ sophisticated multi‐tier defense strategies encompassing SL biosynthesis, lignin deposition enhancement, hypersensitive cellular responses, and hormone‐coordinated immunity. Key discoveries, such as receptor kinases and horizontal gene transfer events, highlight evolutionary arms races between parasites and hosts. Emerging technologies like CRISPR offer promising avenues for engineering resistant crops by disrupting parasitic signaling or enhancing host immunity. This review underscores the importance of integrating molecular insights with agricultural innovation to mitigate yield losses and addresses future challenges, including climate‐driven parasite spread and the need for sustainable, genomics‐driven solutions. By deciphering the silent dialogue between parasites and hosts, this work provides foundations for transformative strategies to safeguard global food security.

## Graphical abstract

Parasitic plants devastate global agriculture through sophisticated molecular interactions with host crops. This review synthesizes current understanding of parasitic plant‐host interactions, from strigolactone‐mediated germination and haustorium formation to host defense mechanisms. We discuss integrated resistance strategies combining genetic engineering, chemical interventions, and ecological management, addressing critical knowledge gaps to advance sustainable control of these agricultural threats.

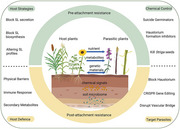

## 1 Introduction

Inter‐organism communication represents an essential life‐sustaining process throughout natural ecosystems. Interspecies symbiotic associations within the plant kingdom manifest as mutualistic, competitive, or parasitic interactions [1]. These plant‐plant interactions play critical roles in shaping ecosystems, but severely affect food crop production worldwide. For instance, the parasitic plant *Striga hermonthica* has been identified as one of the seven major biological threats to food security [2]. Annually, approximately 70 million hectares of agricultural land are infested by this parasite, endangering the food security of nearly 300 million people worldwide [3]. Global food scarcity has emerged as a pressing challenge due to increasing population growth, making stable and enhanced grain yields an urgent priority. Therefore, the substantial agricultural losses caused by parasitic plants have driven extensive research into the interaction between parasitic plants and crops over the past decade, with the goal of mitigating the devastating impact of parasitism on crop yields [3, 4].

In recent years, the threat posed by parasitic plants has intensified significantly, driven by multiple converging factors that demand urgent attention. Climate change has emerged as a primary driver, creating more favorable conditions for parasitic plant proliferation and geographic expansion—rising temperatures and altered precipitation patterns have extended the growing seasons and geographic ranges of many parasitic species, particularly *Striga* and *Orobanche*, enabling them to colonize previously inhospitable regions [5, 6, 7]. Simultaneously, intensified global agricultural trade and seed exchange have inadvertently facilitated the intercontinental dissemination of parasitic plant seeds, introducing devastating species to new agricultural ecosystems that lack natural resistance mechanisms. Most critically, the social and economic impact has become increasingly severe, with smallholder farmers in sub‐Saharan Africa bearing the heaviest burden—crop yield losses of 40%–100% translate to food insecurity for over 100 million people annually, perpetuating cycles of poverty and undernutrition in regions already vulnerable to climate‐driven agricultural challenges. These intersecting pressures underscore the critical need for innovative, sustainable, and scalable solutions to combat parasitic plant infestations, making research into plant‐parasite interactions not merely academically important, but essential for global food security and agricultural sustainability in an era of unprecedented environmental and demographic change.

**FIGURE 1:**
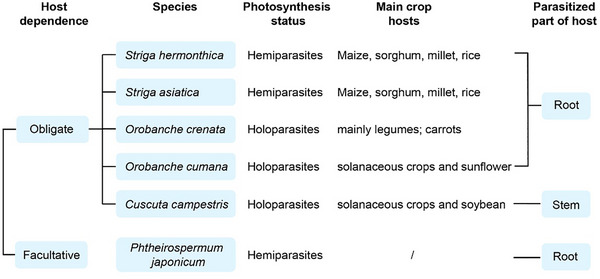
Some of the most extensively studied important parasitic plants. These significant parasitic plants are categorized based on Host dependence, Photosynthesis status, and Parasitic parts. For example, *Striga hermonthica* is classified as an obligate root hemiparasite, while *Cuscuta campestris* is an obligate stem holoparasite. The final column lists the primary hosts of these parasitic species.

Parasitic plants comprise a substantial ecological community, with approximately 4530 among 369 000 flowering plant taxa (1.2%) exhibiting parasitic characteristics, with parasitism having evolved independently across at least 12 angiosperm lineages [8]. Based on host dependence, parasitic plants are classified as obligate parasites (requiring hosts to complete their life cycles) or facultative parasites. According to photosynthetic status, parasitic plants are further categorized as hemiparasites (photosynthetic) or holoparasites (non‐photosynthetic). Finally, based on attachment sites, they are divided into root parasites and stem parasites (Figure 1). For example, members of the orobanchaceous parasites, such as *Phelipanche* and *Orobanche*, function as obligate root holoparasites. Beyond the Orobanchaceae, dodder (*Cuscuta* spp.) and mistletoe (*Viscum* spp.) represent obligate stem parasites, with dodder being holoparasitic and mistletoe hemiparasitic [3, 9].

Historical documentation of parasitic plant emergence dates to approximately 2nd century BCE, and subsequently, these plants achieved global distribution and caused severe agricultural damage. Economically devastating impacts are frequently reported, with Orobanchaceae representatives, particularly *Striga* (witchweeds) and *Orobanche* (broomrapes), ranking among the most agronomically destructive parasitic plants worldwide [10, 11]. *Striga asiatica* and *S. hermonthica* constitute the major witchweed groups causing significant constraints to crop productivity. Both species originated in Africa and exhibit similar host ranges. Major African crops, including sorghum, millet, and maize, are recognized as hosts by *S. asiatica* and *S. hermonthica* (Figure 1), with grain yields typically reduced by 40%–100% due to parasitism, resulting in losses exceeding US$1 billion annually [4, 12]. Broomrapes such as *Orobanche crenata* and *Orobanche cernua* are distributed throughout Europe, the Middle East, and China. In these regions, broomrapes constitute serious threats causing significant economic losses annually [13]. *O. crenata* primarily targets faba bean (*Vicia faba* L.), with over 50% of faba bean cultivation areas experiencing infestation in Spain, Portugal, Syria, and Morocco, resulting in yield reductions of 30%–50%. *Orobanche cumana* severely affects sunflower‐producing regions in central and eastern Europe and has recently expanded into East Asian countries like China, causing serious yield losses in sunflower production in Inner Mongolia in recent years [14].

This review synthesizes recent advances in understanding parasite‐host interactions, with a particular focus on the life cycle of parasitic plants, mainly including orobanchaceous parasite and dodder, and their interactions with hosts at each stage. It summarizes the multiple mechanisms by which hosts have evolved to resist parasitic plant invasion, including alterations in host chemistry and metabolism, as well as reliance on immune‐like responses. Additionally, we discussed the contributions of biotechnological approaches to controlling parasitic plant invasion. We tried to provide insights into novel and effective methods for managing parasitic plant threats for crop production.

## 2 The Life‐Cycle of Parasitic Plants and Interaction with Hosts

### 2.1 Seed Germination

**FIGURE 2:**
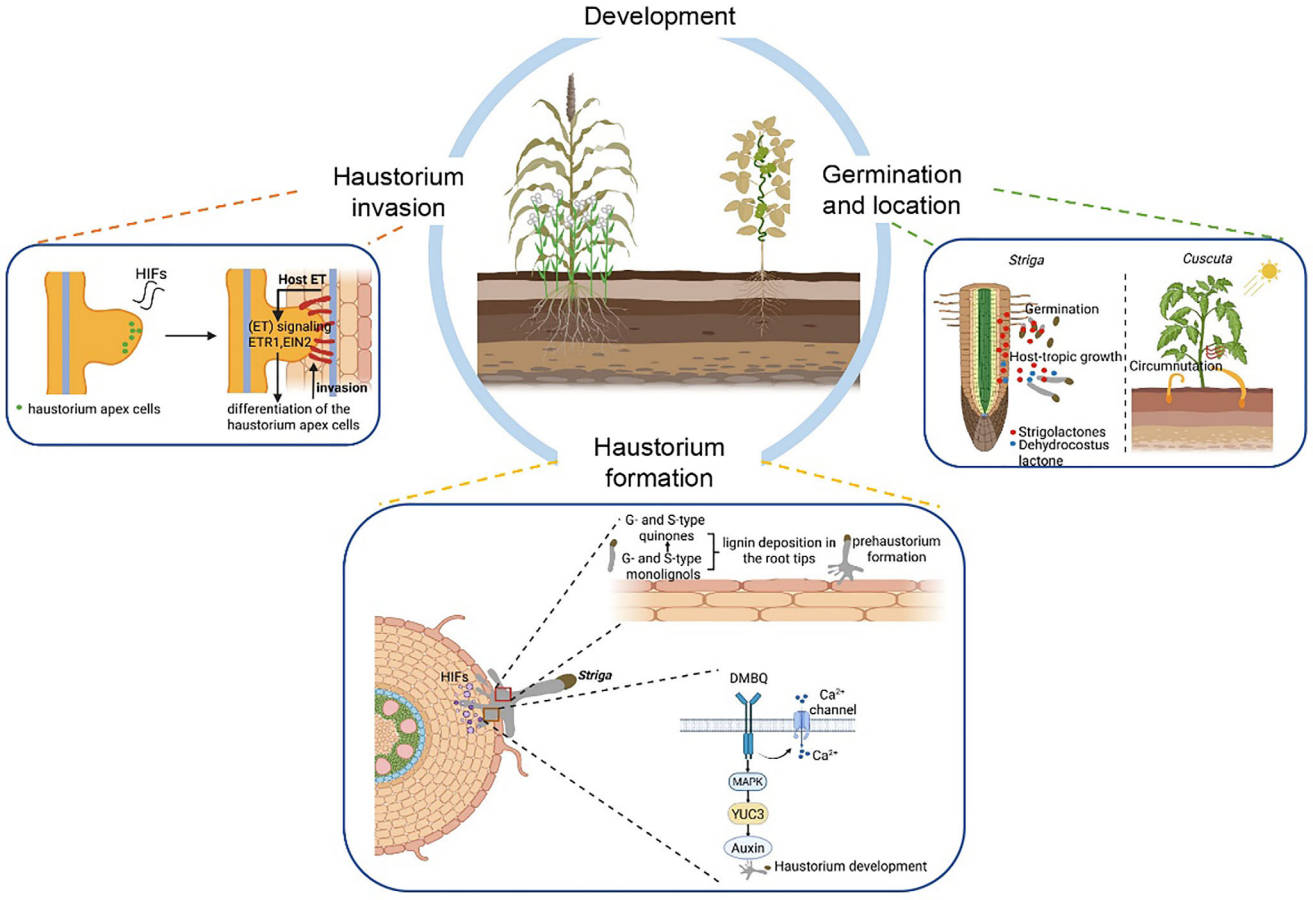
Germination and host‐derived growth strategies of parasitic plants. Root‐parasitic species such as *Striga* rely on strigolactones (SLs) to trigger seed germination. These plants then grow toward host roots using SLs and dehydrocostus lactone as host‐derived chemoattractants. Stem‐parasitic plants like *Cuscuta* do not require chemical signals for germination. Instead, their seeds undergo physical scarification to break dormancy. Post‐germination, the seedlings exhibit circumnutation (helical growth) and orient toward host stems guided by light gradients and volatile organic compounds (VOCs). Haustorium induction is driven by host factors, where host‐derived G‐ and S‐type monolignols are simultaneously incorporated into the cell wall lignin matrix to promote prehaustorium formation. Cntral to this process is the perception of DMBQ signaling via CARD1. The perception of quinones by CARD1 initiates calcium ion influx, and activates MAPK signaling pathways, driving the expression of defense‐associated genes. Functional orthologs of CARD1, including SaCADL1 in *Striga hermonthica* and CADL1, 2, 3 in *Phtheirospermum japonicum*, demonstrate conserved roles in quinone sensing, as evidenced by their ability to rescue quinone recognition defects in *card1/hpca1* mutants. As the hastorium develops, ethylene (ET) signaling in parasitic plants critically modulates the progression from primordia emergence, which is triggered by HIF perception, to the transformation of apical cells into invasive structures. This ET‐mediated orchestration not only facilitates parasitic penetration but also enhances host susceptibility to parasitic invasion.

Parasitic plants in the Orobanchaceae family, including *Orobanche*, *Phelipanche*, and *Striga*, depend on specific signals to break seed dormancy and initiate germination (Figure 2) [15]. In 1823, Vaucher first documented that parasitic plant seeds require external stimuli to germinate. The first germination stimulant for the root parasite *Striga lutea* Lour, named strigol, was isolated from cotton root exudates in the twentieth century [16]. Strigol was subsequently detected in root exudates of sorghum (*Sorghum bicolor* (L.) Moench), maize (*Zea mays* L.), and other crops [17]. Strigol is also discovered as the first type of strigolactones (SLs). Over the past two decades, SLs were also found to play instrumental roles in regulating plant architecture and enhancing arbuscular mycorrhizal (AM) fungal symbiosis [18, 19, 20]. However, the original function of SLs has remained questionable. Recent studies identified bryosymbiol (BSB), a novel SL type from *Marchantia paleacea*. BSB‐deficient mutants exhibit impaired AM symbiosis, but have little effect on the growth of *M. paleacea*. Importantly, *M. paleacea* was found to fail to perceive BSB due to lacking the cognate SL receptors, which implying that it synthesizes SLs for secretion into the rhizosphere to facilitate AM symbiosis [21]. However, these compounds are recognized by orobanchaceous parasites as germination stimulants, which causes the parasitism disaster.

Orobanchaceous parasites can utilize an analogous yet evolutionarily ancestral mechanism involving KARRIKIN INSENSITIVE 2 (KAI2), a homolog of host SL receptor D14, to sense SLs. For example, *S. hermonthica* detects exogenous SLs via receptors in the KAI2d subclade (also termed HYPOSENSITIVE TO LIGHT, HTL) [22, 23, 24]. While KAI2 was initially identified in non‐parasitic *Arabidopsis* as a receptor for karrikins, smoke‐derived germination stimulants, orobanchaceous parasites have expanded the KAI2d subclade through rapid evolution [25]. These *KAI2d* variants possess expanded ligand‐binding cavities that can accommodate SLs [23, 24]. Notably, the *S. asiatica* genome encodes 21 KAI2 paralogs, with 17 belonging to the KAI2d subclade, along with 7 KAI2 pseudogenes [26]. The frequent genomic colocalization of KAI2 paralogs and pseudogenes indicates ongoing dynamic local duplication events within this gene family. Whether distinct KAI2d receptors exhibit differential responsiveness to various SL types remains unresolved. If confirmed, this functional diversification would suggest adaptive evolution of KAI2d receptors for recognizing diverse host‐derived SLs.

In contrast to *Orobanche* (broomrape) and *Striga* (witchweed), the seed germination of the stem parasite *Cuscuta* spp. (dodders) does not require chemical induction from host root exudates [27]. Their dormancy mechanism originates from a physical barrier, the impermeable and structurally dense seed coat [28]. This characteristic confers remarkable resistance even to soil solarization treatments. It persists until seed coat scarification occurs through soil microbial activity or agricultural tillage practices (Figure 2) [29]. The presence of secondary dormancy, a phenomenon prevalent in the germination cycles of numerous weed species, remains undetermined in *Cuscuta* spp. [30]. Additionally, temperature‐dependent regulation of spring germination has been documented, whereas data on seed longevity, emergence capacity across different burial depths, and post‐storage viability remain to be explored.

### 2.2 Host Location and Directed Growth of Parasitic Plants

Although seed germination represents the critical initial phase of parasitism, the limited endogenous nutrient reserves in parasitic plant seeds necessitate that both root parasites (*Orobanche* and *Striga* spp.) and stem parasites (*Cuscuta* spp.) rapidly detect and locate their hosts to establish successful infections. The chemotropic growth of parasitic plant germ tubes has long been hypothesized to serve as the mechanistic basis for this rapid host detection [1].

Recent breakthroughs have revealed that SLs also act as host‐derived chemoattractants in the chemotropic growth of root parasitic plants [31]. Chemotropic assays confirmed SL‐mediated directional growth responses in the obligate parasitic species *S. hermonthica*, while being completely absent in non‐parasitic species. Mechanistic characterization revealed that *Phtheirospermum japonicum* encodes putative receptors for exogenous SL perception, with expression of dominant‐negative receptor variants significantly attenuating this chemotropic capacity. Additionally, dehydrocostus lactone exuded by sunflower (*Helianthus annuus*) was revealed as another potent chemotactic agent that induces directional germ tube growth of *O. cumana* at micromolar concentrations [32]. Notably, GR24 (synthetic SL analog) failed to elicit comparable chemotropic responses in *Orobanche* parasitism, demonstrating functional specificity of chemoattractants in different parasitic plants (Figure 2) [32].

In fact, due to inherent challenges in observing subterranean growth of root parasites, *Cuscuta* (dodder) has emerged as the primary model system for investigating host location and tropic growth mechanisms in parasitic plants. Following seed germination, *Cuscuta* seedling stem initiates host‐seeking behavior via circumnutational movement; the regulatory mechanisms of this behavior in response to diverse environmental signals have been comprehensively investigated (Figure 2) [33, 34, 35, 36]. Volatile organic compounds (VOCs) constitute a critical factor mediating *Cuscuta* tropism toward host plant stems (Figure 2) [37, 38]. In controlled environments, *Cuscuta pentagona* seedlings were found to grow toward tomato plants and purified tomato volatiles, and similar chemotropic responsiveness was detected in response to VOCs from wheat and impatiens. Notably, these seedlings display discriminative chemoperception. They preferentially growing toward tomato‐derived volatiles over wheat‐derived ones, with specific individual compounds inducing tropic growth while others exhibit repellent effects [37].

Light signals also play critical roles in host location by *Cuscuta*. *Cuscuta campestris* seedlings exhibit a significant acceleration in circumnutation rates under high red/far‐red (R/FR) light ratios, indicating the involvement of phytochrome signaling pathway [39, 40, 41, 42]. Multiple *Cuscuta* species displayed far‐red light‐directed preferential stem elongation, which shows convergence with shade avoidance responses. In contrast, host‐stem coiling behavior is synergistically induced by blue and far‐red light, whereas it is significantly inhibited by red light irradiation [42, 43, 44, 45, 46, 47, 48].

Collectively, root parasites (*Orobanche* and *Striga* spp.) and stem parasites (*Cuscuta* spp.) have evolved distinct host location strategies, shaped by their unique habitats and target host tissues. Confined to subterranean environment, root parasites employ a chemically dependent, sessile approach: post‐germination, they lack motile structures and rely exclusively on host‐secreted small molecules to direct germ tube chemotropism toward roots, with species‐specific chemoattractant preferences. In contrast, aerial‐dwelling *Cuscuta* uses a dynamic, multimodal strategy: it employs circumnutation to scan its surroundings, integrates host‐derived VOCs and light signals for guide directional growth, and utilizes light‐dependent coiling for host attachment. These divergent strategies underscore the evolutionary adaptability of *Cuscuta* to their ecological niches. Notably, despite their distinct parasitic strategies—root vs. stem parasitism—both orobanchaceous parasites and *Cuscuta* spp. rely on chemical signals for host detection and directional growth, suggesting the existence of conserved mechanisms underlying chemotropism across diverse parasitic plant lineages.

### 2.3 Haustorium Formation and Invasion of Host Plant

Upon contact with host plants, specific host‐derived compounds induce haustorium formation in orobanchaceous parasites [49]; this host‐derived compound‐dependent induction, however, is not required for *Cuscuta* spp. to accomplish the same process. Haustoria development establishes vascular connectivity between parasite and host, enabling bidirectional resource translocation. This haustorium formation process in orobanchaceous parasites is mediated by a suite of phytochemicals, including 2,6‐dimethoxy‐1,4‐benzoquinone (DMBQ), flavonoids, and phenolic compounds, which are collectively defined as haustorium‐inducing factors (HIFs) [50, 51, 52]. These HIFs share a conserved redox‐active methoxyphenol moiety. Methoxyphenols and methoxyquinones likely originate from host cell wall lignin via two pathways: peroxidase‐mediated oxidation of lignin degradation products, or as byproducts of lignin polymerization [51, 53].

Recent studies revealed that exposure to HIFs triggers lignin deposition in the root tips of *S. hermonthica*, with host‐derived G‐ and S‐type monolignols being simultaneously incorporated into the cell wall lignin matrix to promote prehaustorium formation (Figure 2) [54]. HIFs significantly upregulate the activity of enzymes involved in monolignol biosynthesis and polymerization, and inhibition of these enzymes impairs prehaustorium development [54].

Among HIFs, DMBQ—first isolated from sorghum root exudates—remains the most potent inducer, stimulating haustorium formation across multiple orobanchaceous parasites [52, 55]. DMBQ perception was found to be mediated by a leucine ‐rich‐repeat receptor like kinase, CANNOT RESPOND TO DMBQ1 (CARD1), which was identified via a forward genetic screen of DMBQ‐unresponsive *Arabidopsis* mutants [56]. *P. japonicum CARD1* knockdown lines fail to form functional haustoria. Crucially, reactive oxygen species (ROS), particularly hydrogen peroxide (H_2_O_2_), are essential for haustorium induction [57], and CARD1 deficiency also disrupts ROS‐mediated signaling required for haustoria development, which together indicating CARD1 was the bona fide DMBQ receptor governing the formation of haustorium in orobanchaceous parasites (Figure 2).

Moreover, while DMBQ effectively induces haustorium formation in *P. japonicum* and *S. hermonthica*, *Orobanche* and *Phelipanche* species respond poorly to DMBQ but are induced by *Brassica napus* root exudates or fungal metabolites like sphaeropsidone [58, 59]. It indicated that members of the orobanchaceous parasites exhibit species‐specific responsiveness to HIFs. This functional diversity implies multiple molecular pathways govern haustoria induction across parasitic lineages.

Following HIF‐mediated host attachment, orobanchaceous parasites undergo a coordinated developmental program involving epidermal and cortical cell redifferentiation at the root contact site, forming swollen protrusions that secrete adhesive molecules for host anchorage [50, 60]. This process is orchestrated by auxin signaling, with YUC3‐mediated auxin biosynthesis triggering haustorium initiation at the root transition zone and coordinating cell division during early infection [61, 62, 63, 64]. Subsequently, specialized invasive cells differentiate at the haustoria apex, exhibiting elongated morphology and palisade‐like alignment adjacent to host cells, with their formation regulated by SUBTILISIN‐LIKE SERINE PROTEASES (SBTs) and ethylene signaling components including ETR1 and EIN2 (Figure 2) [65, 66, 67]. These invasive cells successfully penetrate host tissues and, along with adjacent xylem‐proximal stem cells, differentiate into tracheary elements that interconnect through capillary fusion to form functional xylem bridges (XB), establishing the vascular continuity essential for resource translocation [65, 68]. Notably, while hemiparasites like *S. hermonthica* and *P. japonicum* rely primarily on xylem connections for nutrient acquisition, holoparasites such as *Orobanche* species have evolved additional phloem connectivity, compensating for their photosynthetic loss through enhanced resource extraction capabilities [69, 70, 71].

### 2.4 Host‐Parasite Biomolecule Exchange: From Nutrients to Horizontal Gene Transfer

Following xylem bridge formation, orobanchaceous parasites initiate resource extraction through host vascular connections. Nutrient translocation in *Striga* species and *P. japonicum* predominantly occurs via xylem continuity [69, 71], while *Orobanche* lineages additionally exploit phloem connectivity for bidirectional metabolite exchange [70]. Orobanchaceous parasites exhibit differential ion accumulation strategies, preferentially uptaking cations such as potassium and synthesizing osmolytes like mannitol to generate osmotic gradients driving water influx [72, 73, 74, 75]. Carbon acquisition increases steadily from facultative—which get about 10% of their carbon from host—to obligate hemiparasites (∼30%) and reaches its peak in holoparasitic species, which rely almost entirely on hosts for carbon. This pattern reflects an evolutionary gradient in how parasites exploit nutrients [76].

Beyond primary metabolites, parasitic plants take up host‐synthesized secondary metabolites via vascular connections to the host. For example, the hemiparasite *Castilleja indivisa* cannot produce alkaloid on its own, but instead actively acquires the alkaloid lupanine from its host plant, *Lupinus texensis* [77]. Similarly, the hemiparasite *Rhinanthus serotinus* sequesters mycotoxins from its host *Lolium pratense* [78], and the species of the parasitic genus *Cuscuta* hyperaccumulate glucosinolates from their *Arabidopsis* hosts. Notably, *Cuscuta*, glucosinolates concentrations in its tissues surpass those in the host—and these compounds play critical roles in mediating the parasite's ecological interactions with insect herbivores [79, 80, 81].

Parasitic plants and their hosts also interchange genetic materials, including RNAs and proteins, which can influence their interspecific interactions [3]. For example, after *Cuscuta pentagona* parasitizes *Cucurbita maxima*, eight host‐derived mRNAs were detected to be translocated into the parasite [82]. Further study of the *C. pentagona*‐*Solanum lycopersicum* (tomato) system revealed 474 tomato‐originated transcripts within dodder tissues [83]. Functional RNA‐interference (RNAi) signals also move across the haustoria of parasitic plants. One study engineered an RNAi system in tobacco to target the transcription factor SHOOT MERISTEMLESS‐like (STM) in *C. pentagona*, and this resulted in significantly reduced biomass of the dodder growing on the transgenic tobacco hosts [84]. Similarly, using virus‐induced gene silencing (VIGS) to target specific genes in *O. cumana*, including *OcQR1*, *OcCKX5*, and *OcWRI1*, led to a marked decrease in the number of haustoria it produced [85]. Beyond mRNAs and small RNAs, recent proteomic studies have revealed extensive protein trafficking between parasitic plants and their hosts. In *Cuscuta*‐host (*Arabidopsis* and soybean) systems, hundreds to more than 1500 proteins are bidirectionally transferred, accounting for a few to over 10% of the foreign plant's proteome. These mobile proteins have been confirmed to retain biological activity, and the inter‐plant protein translocation could be propagated into seeds of both soybean and dodder [86]. Notably, in terms of abundance, mobile proteins are far more prevalent than mobile mRNAs (6.8% vs. 0.2%) in the soybean‐dodder parasitism system, implying that proteins may serve as the primary form of interplant molecular communication [87]. Furthermore, most detected mobile proteins were not *de novo* synthesized from the translocated mRNAs, but are bona fide mobile proteins [86]. In addition, this interplant exchange of macromolecules is dynamically regulated by environmental conditions such as nutrient stress [87].

Transcriptomic and genomic data have confirmed horizontal gene transfer (HGT) between parasitic plants and their hosts [26, 88, 89, 90]. Orthologs of the *S. hermonthica* gene *ShContig9483* show phylogenetic conservation only within Poaceae species, including its natural host *S. bicolor* [88]. Moreover, *S. asiatica* retains *ShContig9483* and maintains synteny (genetic order) of the flanking host‐derived loci. Notably, conservation of both intron structure and untranslated regions (UTRs) suggests that *S. asiatica* acquired substantial genomic segments (∼30 kb) via DNA‐mediated HGT mechanisms [26]. This pattern is consistent across the orobanchaceous parasites, where HGT events containing introns are most common, strongly supporting genomic DNA transfer as a major route of inter‐species genetic exchange [89, 90, 91, 92]. Within parasitic plants, *Cuscuta* species exhibit unusually high genomic DNA‐based HGT frequencies, and their transfer rates are significantly higher than other parasitic lineages [93].

In short, biomolecule transfer between parasitic plants and hosts is fundamental to parasitism and coevolution. Vascular links enable uptake of primary nutrients, with carbon reliance scaling by parasitic type, and host secondary metabolites that probably enhance parasite fitness and herbivore defense. Moreover, the functional RNAs, proteins, and HGT from host to parasitic plants drive parasitic evolution and adaptation. These exchanges shape host‐parasite specificity, informing the resistance mechanisms discussed next.

## 3 Host Plant Resistance Mechanisms against Parasitic Plants

Host plant defense strategies are closely coordinated with the parasitic plant life cycle, targeting specific stages of parasitism to prevent successful infection. Host resistance mechanisms primarily focus on disrupting key stages, including seed germination, host detection, haustorium formation, and invasion. Based on the timing of defense deployment relative to physical contact, these mechanisms can be categorized as pre‐attachment resistance (acting during seed germination and host location stages before parasite attachment) or post‐attachment resistance (acting following physical contact, during haustorium formation, invasion, and resource extraction stages). Pre‐attachment resistance includes strategies to reduce parasitic seed germination, block haustorium formation, or render hosts “invisible” to parasites. Post‐attachment resistance encompasses host recognition of parasites, hormone‐mediated defense responses, cell wall modifications, and immune‐like reactions that limit parasitic establishment and resource extraction.

### 3.1 Pre‐Attachment Resistance

**FIGURE 3:**
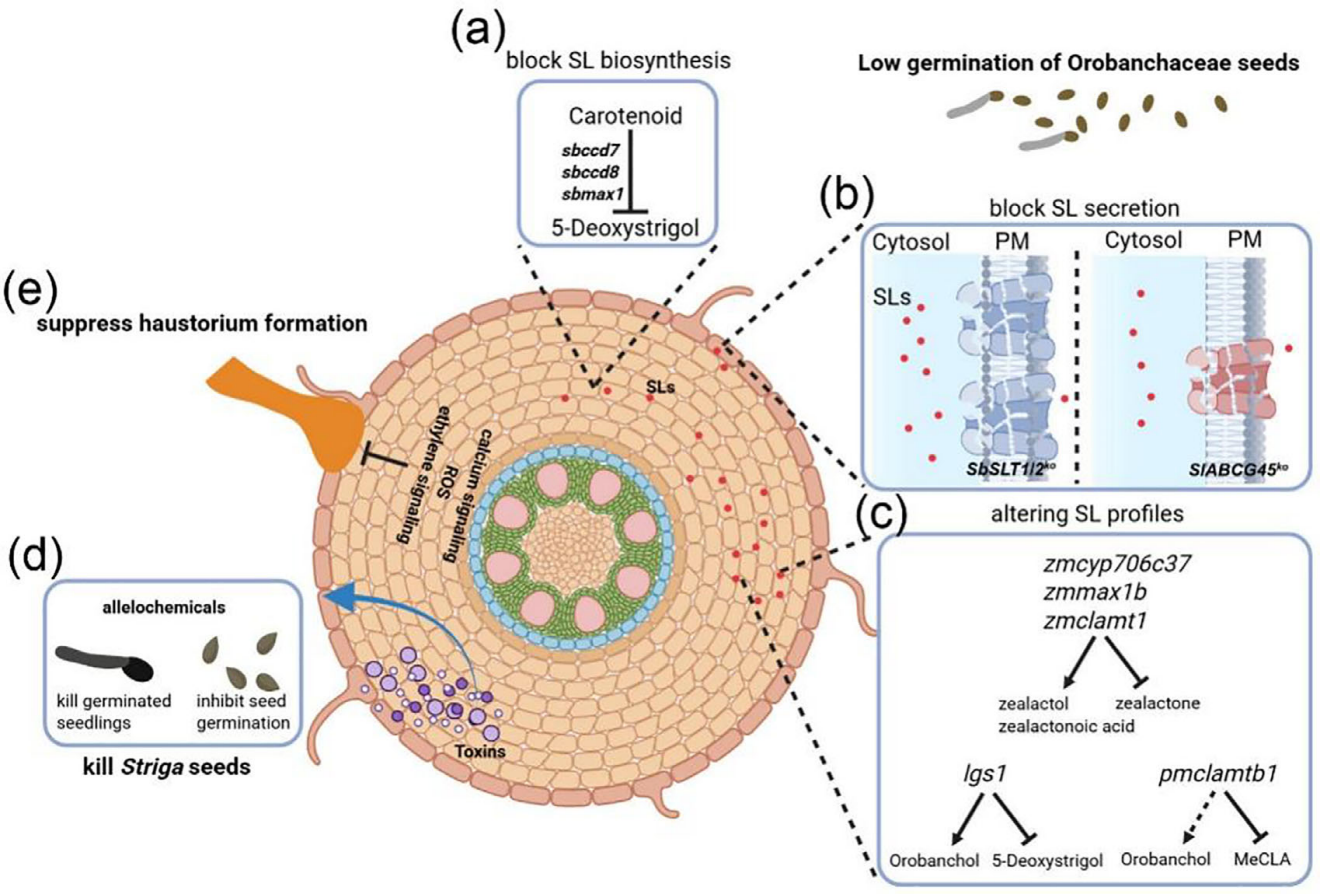
Host pre‐attachment resistance to parasitic plants. (a)‐(c) Pre‐attachment resistance mechanisms targeting the strigolactone (SL) pathway involve disrupting SL biosynthesis and transport. SL synthesis suppression through knockout of CCD7, CCD8, and MAX1 (a), SL export blockade by disrupting SL transporters (SLT1/SLT2 in sorghum; ABCG45 in tomato) (b), and SL profile modification in root exudates via LGS1 mutations in sorghum, CYP706C37/MAX1B/CLAMT1 mutations in maize, and CLAMT1B knockout in millet (c), These genetic interventions collectively inhibit parasitic seed germination by altering SL‐mediated chemical communication. (d) Sunflower (*Helianthus annuus*) cultivars with resistance to *Orobanche cernua* synthesize and accumulate phytotoxic phenolics—notably 7‐hydroxylated coumarins such as scopoletin and ayapin (purple highlight)—during parasitic infection. These specialized metabolites act as germination inhibitors and seedling growth suppressants, disrupting *O. cernua* establishment. (e) Haustorial development disrupted by reactive oxygen species (ROS) production, ethylene signaling, and calcium signaling.

As described above, orobanchaceous parasites germination always depends on SLs, indicating that host‐derived SL levels may determine whether parasites successfully germinate and establish parasitism. The most direct approach to prevent orobanchaceous parasites germination involves disrupting SL biosynthesis in host plants. Mutations in *CAROTENOID CLEAVAGE DIOXYGENASE 8* (*CCD8*) can abolish SL production in sorghum, and thus root exudates from *ccd8* mutants significantly reduce *Striga* seed germination compared to wild‐type exudates [94]. Similarly, the sorghum *NONDORMANT AXILLARY BUD 1* (*NAB1*) gene, which encodes a *CAROTENOID‐CLEAVAGE DIOXYGENASE 7* (CCD7), has been mutated to impairing SL biosynthesis, and the hydroponic solutions of *nab1* mutants suppress *Orobanche* seeds germination (Figure 3a) [95]. However, manipulating SL pathways carries physiological trade‐offs. SL deficiency disrupts endogenous plant development, causing dwarfism, excessive tillering, and other growth abnormalities that compromise agricultural productivity [95]. Thus, identifying strategies to modulate SLs or other stimulants of parasitic seed germination, while maintaining the balance between normal plant growth and resistance to parasitic plants, remains a critical challenge.

In addition to reducing SL biosynthesis, another effective strategy entails blocking SL secretion into the rhizosphere, while preserving physiological SL levels within the plant to sustain their endogenous regulatory functions. In petunia (*Petunia hybrida*), PDR1 was identified as the first SL transporter in dicots [96]. Mutation of *pdr1* significantly reduced orobanchol levels in root exudates, leading to markedly lower germination rates of *P. ramosa* seeds when treated with mutant exudates [96]. Subsequent studies identified SL transporter in alfalfa (*Medicago truncatula*), MtABCG59, which is a PDR1 homolog [97], However, these earlier studies did not address whether inhibiting SL efflux in crop hosts of orobanchaceous parasites, such as *Striga* and *Orobanche* could reduce germination rates of the parasites and thereby mitigate crop yield losses.

Two recent studies have resolved this critical gap. Two SL transporters, SbSLT1 and SbSLT2, have been identified in sorghum via integrated RNA‐seq analysis of Pi deficiency and GR24^5DS^ treatment [98]. Knockout of these transporters in sorghum drastically reduced 5‐deoxystrigol (5DS) levels in root exudates, suppressing *Striga* seed germination. Field trials demonstrated that mutant plants maintained normal growth while harboring significantly fewer *Striga* infestations, resulting in substantially reduced yield losses (Figure 3b) [98]. AI‐driven molecular simulations further revealed conserved amino acid residues essential for SL transport, highlighting mechanistic conservation across monocot and dicot SL transporters [98]. In addition, the ZmSLT1 and ZmSLT2 also were proved to possess the SL exporter activity. In parallel, two SL transporters, SlABCG44 and SlABCG45, were identified in tomato through forward genetic mapping. Knockout of either gene effectively blocked SL secretion and reduced *Orobanche* germination (Figure 3b). Interestingly, knockout of *SlABCG44* negatively affected fruit size, while knockout of *SlABCG45* had minimal impact. This contrasts with the findings in sorghum, where individual knockout of either *SbSLT1* or *SbSLT2* showed no adverse effects on agronomic traits [99]. Two‐year field trials confirmed that *SlABCG45* knockout lines had fewer parasitic attachments and lower *Orobanche* biomass, while tomato fruit size remained unchanged, and yield per plant increased significantly [100]. Both studies conclusively demonstrate that disrupting SL transporter activity effectively enhances host resistance to orobanchaceous parasites without compromising plant growth, establishing this approach as a feasible agricultural strategy.

Since seeds of orobanchaceous parasites typically require recognition of specific SL conformations for germination [101], altering the conformation of SLs synthesized by plants to prevent seed germination may represent a precise strategy for parasitic resistance [102]. Several genes involved in altering the conformation of SLs synthesized by plants have been identified, and these genes are closely associated with orobanchaceous parasites resistance. Mutation in the *LOW GERMINATION STIMULANT 1* (*LGS1*) gene confers significant resistance to *Striga* in sorghum (Figure 3c). *LGS1* encodes a sulfotransferase, and following the mutation, the main types of SLs synthesized in sorghum roots were altered [12]. The 5DS form represents the dominant SL type synthesized in sorghum, which strongly induces *Striga* seed germination and parasitism. In the *lgs1* mutant, the primary SL synthesized in sorghum roots shifted to the orobanchol form, which significantly reduced its capacity to stimulate *Striga* seed germination [12]. It is noteworthy that while orobanchol is not a common SL type in sorghum, it is still capable of fulfilling the SL‐mediated functions essential for the normal growth and development of the plant.

In *Pennisetum glaucum* (pearl millet), comparing SL profiles in root exudates of the *Striga*‐susceptible line P10 and resistant line Aw revealed that the susceptible line P10 secretes four distinct SLs, which are entirely absent in Aw. Comparative genomic analysis identified a 0.7 Mb chromosomal deletion in the Aw genome encompassing two putative *CARLACTONOIC ACID METHYLTRANSFERASE1* (*CLAMT1*) genes [103]. Functional validation confirmed that *CLAMT1b* in pearl millet catalyzes carlactonoate (MeCLA) production, which is the precursor of P10‐specific SLs. Population‐level screening across a diverse pearl millet panel conclusively demonstrated that the *CLAMT1* locus serves as the key genetic determinant governing both SL diversity and *Striga* susceptibility (Figure 3c) [103].

A groundbreaking study on maize resistance against orobanchaceous parasites demonstrated that strategic modification of SL biosynthesis confers effective resistance (Figure 3c). Through systematic screening of a maize germplasm collection, two novel SL variants, zealactol and zealactonoic acid, were identified, which exhibit significantly reduced activity in stimulating *Striga* seed germination compared to the predominant maize SL zealactone [104]. Notably, a single cytochrome P450 enzyme, ZmCYP706C37, was found to catalyze sequential oxidative modifications within the maize SL biosynthetic pathway. Crucially, suppression of this enzyme—along with two other pathway components, ZmMAX1b and ZmCLAMT1—induced substantial alterations in SL composition [104]. In rice, functional diversification among different types of SLs has been revealed [105]. Mutants deficient in canonical SLs do not exhibit the aboveground developmental phenotypes typically associated with SL deficiency; instead, they show delayed arbuscular mycorrhizal symbiosis and a significant reduction in the induction of *Striga* seed germination. Moreover, by blocking canonical SL biosynthesis using TIS108, a specific enzyme inhibitor, *Striga* infection was markedly suppressed without compromising normal rice growth [105]. These results indicated that canonical SLs are dispensable for rice morphogenesis and provide a foundation for enhancing crop resistance to parasitic plants through targeted genes editing or chemical intervention.

An innovative chemical intervention strategy entails the use of potent strigolactone (SL) agonists as suicidal germination inducers. Leveraging the obligate reliance of *Striga* and *Orobanche* species on host‐derived SLs for germination, synthetic SL analogs are applied to infested fields (in the absence of host crops) to trigger parasitic seed germination. As no viable host is available post‐germination, the germinated seedlings perish, progressively depleting the soil seed bank. A breakthrough was achieved with sphynolactone‐7, a selective SL agonist triggering *S. hermonthica* germination at femtomolar concentrations (10^−^^1^ ^5^ m) without interfering with crop SL‐dependent processes [24, 106]. Complementary synthetic SL analogs, including methyl phenlactonoates (MP3, MP16) and Nijmegen‐1, have demonstrated reductions in *Striga* emergence in field trials. Notably, emulsifiable concentrate and slow‐release granular formulations have recently been developed, which are specifically tailored to rain‐fed African agriculture [99, 107, 108, 109, 110, 111]. This approach offers strategic advantages by directly targeting soil seed banks, exploiting parasite biology to counter the parasite itself, thereby reducing the development of resistance. However, implementation challenges remain regarding moisture requirements, application timing, and the development of cost‐effective methods accessible to smallholder farmers.

Besides preventing the germination of seeds of orobanchaceous parasites or inducing their suicidal germination, some host plants secrete toxins to inhibit parasite development (Figure 3d) [112, 113]. For example, *O. cernua* seeds that germinate near resistant sunflowers exhibit discoloration symptoms, followed by growth retardation and eventual death. Further study found that the resistant sunflowers secrete 7‐hydroxylated simple coumarins, a class of defensive secondary metabolites, which create a toxic microenvironment for *O. cumana*. These compounds induce rapid browning and necrosis of germinated *O. cumana* seedlings, effectively halting parasitism even after germination.

In addition to disrupting the development of germinated parasitic seedlings, blocking haustorium formation is also a key pre‐attachment resistance mechanism. It has been reported that wild sorghum accessions exhibit reduced capacity to induce *Striga* haustorium formation compared to cultivated varieties [114]. When *Striga* parasitizes the resistant maize line H614D, *Striga* plants remain small with poorly developed vegetative tissues, preventing establishment of effective vascular connections [115]. It has been hypothesized that resistant hosts may secrete inhibitory compounds—such as auxin antagonists—to disrupt haustorial development [116]. However, the mechanisms underlying this resistance remain unclear. As previously mentioned, haustorium development in parasitic plants depends on host‐derived HIFs, which trigger the parasitic infection process. Synthetic inhibitors of HIFs targeting reactive oxygen species (ROS) production, ethylene signaling, auxin transport/activity, and calcium signaling effectively suppress haustorium formation in parasitic plants (Figure 3e) [56, 62, 66]. These findings underscore the essential role of HIF‐triggered signaling pathways in haustorium initiation and suggest that disrupting these pathways can mitigate parasitic invasion. However, whether host plants naturally secrete analogous inhibitors as an evolved pre‐attachment resistance strategy remains to be elucidated.

### 3.2 Post‐Attachment Resistance

#### 3.2.1 Host‐Specific Recognition of Parasitic Plants

**FIGURE 4:**
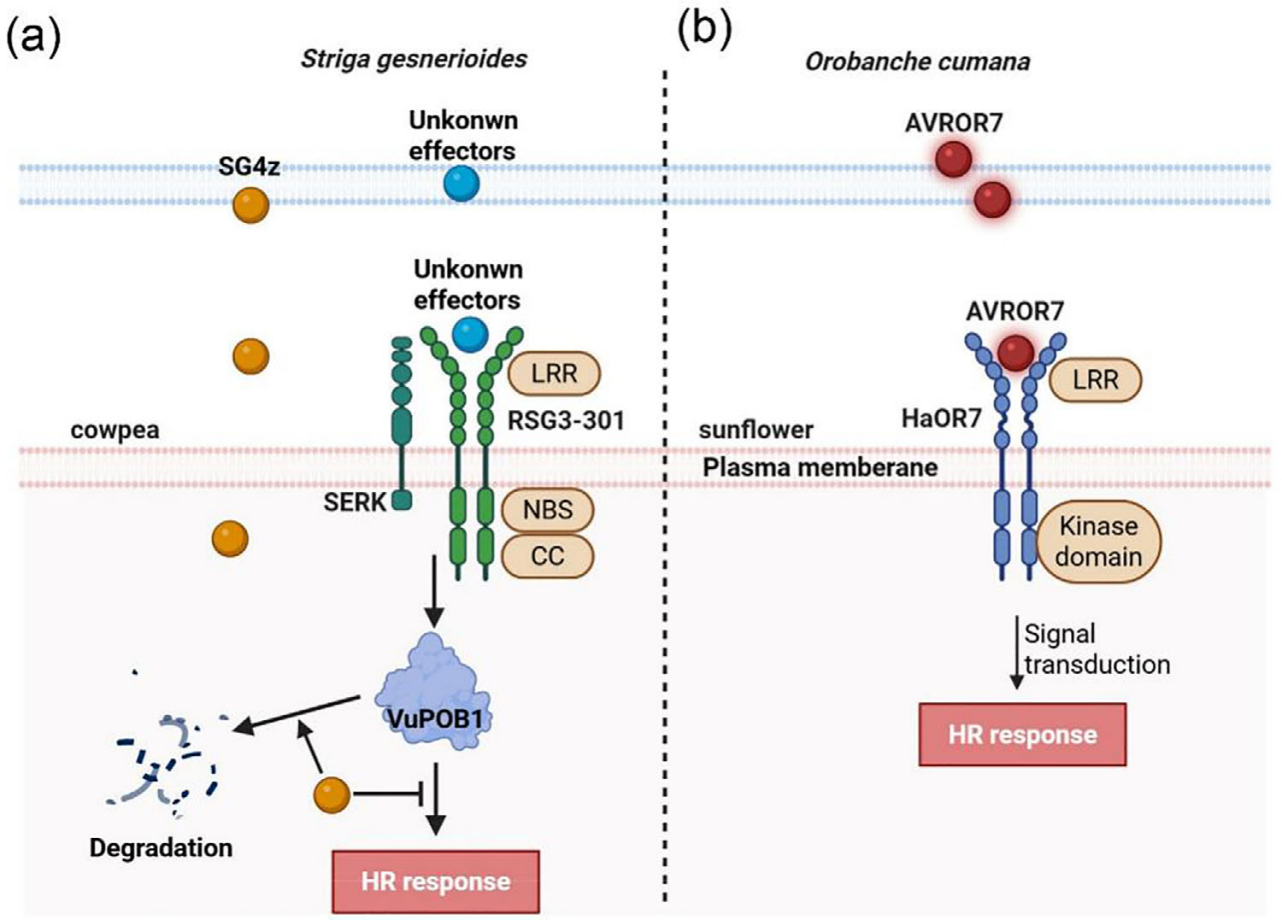
Mechanisms of host pre‐attachment resistance to parasitic plants. (a) In cowpea cultivar B301, infection by *Striga* races SG3/SG4 induces localized cell death (HR) at parasite attachment sites. This defense requires the NLR protein RSG3‐301, which potentially detects *Striga* effectors through association with VuSERK co‐receptors, leading to VuPOB1‐mediated HR potentiation. Conversely, SG4z‐derived effector SHR4z—secreted into the apoplast—translocates into host cells via unidentified transporters. SHR4z‐VuPOB1 binding triggers ubiquitin‐dependent proteolysis of the HR activator, thereby suppressing defensive responses in B301 roots. (b) *Helianthus annuus Orobanche resistance 7* (*HaOr7*) encodes a membrane‐bound leucine‐rich repeat (LRR) receptor kinase. HAOR7 specifically interacts with the *Orobanche cumana* effector AVROR7. This recognition event, which requires collaboration with unidentified cofactors, activates intracellular kinase‐mediated defense signaling. Subsequent hypersensitive cell death responses disrupt *O. cumana* haustorial development by blocking vascular integration into sunflower roots.

Host plants have evolved sophisticated molecular recognition systems to detect and respond to parasitic plant invasion, involving diverse resistance proteins that trigger defense responses. In cowpea (*Vigna unguiculata*), the *RSG3‐301* gene encodes a CC‐NBS‐LRR protein conferring race‐specific resistance to *Striga gesnerioides* race 3 (SG3) [117]. Silencing *RSG3‐301* nearly abolished resistance, demonstrating its critical function in defense against parasitism (Figure 4a). However, the SG4z race has overcome this resistance by secreting SUPPRESSOR OF HOST RESISTANCE 4z (SHR4z), an effector protein that suppresses the host hypersensitive response (HR) [118]. SHR4z interacts with the host E3 ligase *VuPOB1* to accelerate its degradation, thereby suppressing host resistance and enhancing parasitic success [118]. This represents the first identification of a functional effector protein in parasitic plants and highlights the ongoing evolutionary arms race between parasites and hosts.

In sunflower, a membrane‐bound leucine‐rich repeat (LRR) receptor kinase encoded by *H. annuus OROBANCHE RESISTANCE 7* (*HaOr7*) was identified [119]. Resistant sunflower lines express a full‐length HAOR7 protein. In contrast, susceptible lines produce a truncated HAOR7 isoform deficient in both transmembrane anchoring and kinase‐mediated signaling domains, rendering them incapable of initiating defense responses against parasitic invasion [119]. The study revealed that *HaOr7* induces incompatible attachment of *O. cumana* to sunflower roots, effectively blocking vascular connectivity [119]. The discovery of *HaOr7* shows that receptor proteins are essential for blocking parasitic plants attachment and preventing successful infection (Figure 4b).

HR has also been observed during *S. hermonthica* parasitism of other crops. In the rice (*Oryza sativa*) cultivar Nipponbare, distinct HR reactions are triggered upon *Striga* infestation, accompanied by significant induction of HR‐associated proteins [117, 120, 121]. Similarly, in sorghum, localized HR at infection sites in the resistant cultivar IS14963 effectively blocks further *Striga* invasion [122]. However, the molecular mechanisms and specific genes underlying HR against *Striga* in rice and sorghum remain poorly characterized. Analyses of sorghum genotypes with diverse resistance responses to *S. hermonthica* found *Striga* elicits both basal immunity and ETI‐like responses, with resistance linked to genes like a glucan synthase‐like 10, a pathogenesis‐related thaumatin‐like protein, and a phosphoinositide phosphatase—likely involved in ETI‐mediated resistance [123].

**FIGURE 5:**
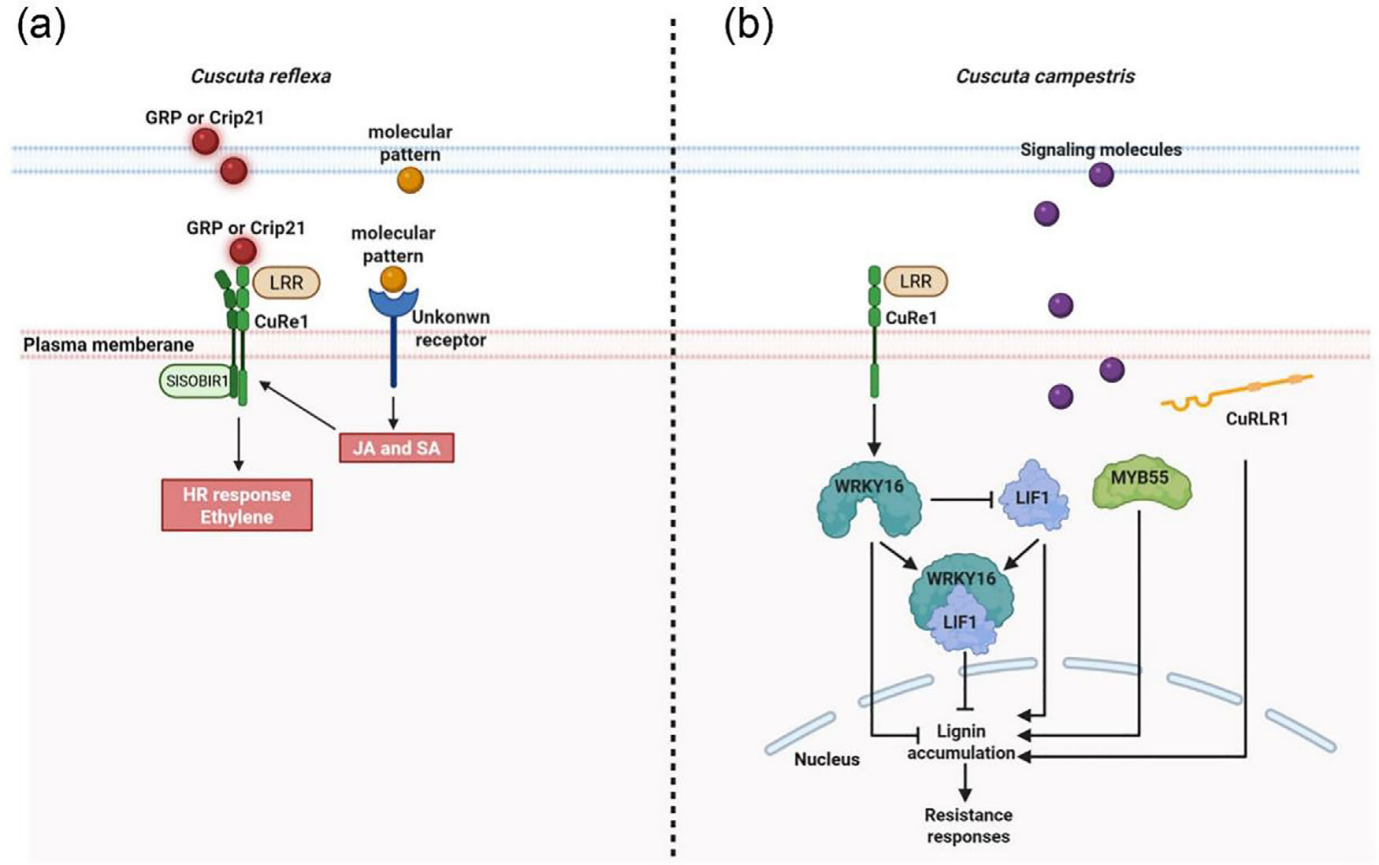
Defense mechanisms in cultivated *Solanum lycopersicum* against the stem parasite. (a) The LRR‐containing receptor‐like protein CuRe1 of host plant detects a 11‐kDa glycine‐rich effector protein (GRP) or its bioactive peptide fragment Crip21, secreted by the parasite *Cuscuta reflexa*. In complex with the co‐receptor SlSOBIR1, CuRe1 activates downstream defense pathways, inducing hypersensitive‐like reactions marked by reactive oxygen species (ROS) bursts and ethylene biosynthesis. A pathogen‐associated molecular pattern from *Cuscuta australis* is detected by an unidentified receptor in tomato, activating jasmonic acid (JA) and salicylic acid (SA) biosynthesis. These phytohormones transcriptionally upregulate *CuRe1*, which coordinates dual defense strategies—both hypersensitive response (HR)‐mediated cell death and non‐HR systemic resistance—against *C. australis* parasitism. (b) The host‐derived receptor‐like protein CuRLR1 potentially senses macromolecular signals from *Cuscuta campestris* to activate downstream pathways that stimulate lignin deposition as a physical barrier. This defense mechanism exhibits hallmarks of Effector‐Triggered Immunity (ETI). Transcriptional activators SlMYB55 and LIF1 drive lignin biosynthesis, while SlWRKY16‐LIF1 regulatory modules may synergize with CuRe1‐mediated signaling to reinforce resistance.

Similarly, host‐specific recognition is evident in interactions between the stem parasitic plant *Cuscuta* and its hosts. CUSCUTA RECEPTOR 1 (CuRe1), a cell surface leucine‐rich repeat receptor‐like protein (LRR‐RLP), has been identified in *C. reflexa* resistance wild tomato (*Solanum chilense*) [124]. Stable transformation of *CuRe1* into *C. reflexa*‐susceptible *S. pennellii* and *N. benthamiana* conferred enhanced resistance to *C. reflexa* infection. Further analysis revealed that tomato resistance to *C. reflexa* may not be solely mediated by *CuRe1*, suggesting that additional pathways likely cooperate with *CuRe1* to achieve full resistance. Subsequently, to identify key factors that cooperate with *CuRe1*, a glycine‐rich protein (GRP) and its minimal peptide epitope Crip21 were identified from parasitic plant cell wall extracts. Both GRP and Crip21 serve as PAMPs, specifically binding to CuRe1 and activating its resistance response (Figure 5a) [125].

#### 3.2.2 Hormone Signaling

Phytohormone signaling pathways play pivotal roles in host plant defense against parasitic plant invasion. Multiple hormones, including salicylic acid (SA), jasmonic acid (JA), abscisic acid (ABA), and ethylene coordinate defense responses, with SA and JA pathways serving as the primary regulators of anti‐parasitic immunity. During the early stages of parasitic infection, JA signaling is rapidly activated, followed by SA pathway induction. For instance, *O. cumana* infection induces JA biosynthesis genes in sunflower roots [126]. During *S. hermonthica* parasitism of rice, JA signaling is activated initially, which is subsequently followed by SA pathway activation [127]. JA‐deficient mutants show increased susceptibility to *S. hermonthica*. This enhanced susceptibility can be restored to normal levels by exogenous JA treatment. Similarly, disruption of the SA pathway also increases plant susceptibility to *S. hermonthica* [127].

Similarly, during *C. pentagona* parasitism of tomato, JA levels peaked 36 h after *C. pentagona* haustorial attachment to the host, while SA accumulation reached its maximum 48 h post‐infection. In the SA‐deficient transgenic tomato line *NahG*, SA is converted to inactive catechol, failing to accumulate SA. Consequently, *NahG* plants are unable to trigger HR and exhibit heightened susceptibility to *C. pentagona* parasitism [128]. *Cuscuta australis* invasion of tomato stems elicits varying degrees of resistance across tomato cultivars, with cultivated tomatoes exhibiting robust HR [129]. Parasitization by *C. australis* significantly induces JA and SA accumulation in tomato stems, and these phytohormones enhance resistance by transcriptionally upregulating *CuRe1* [127]. Notably, JA and SA levels remain normally induced in *CuRe1* mutants during parasitism. These results suggested the existence of an unidentified upstream receptor (Figure 5b) [127]. The findings demonstrate that coordinated JA and SA signaling is essential for effective host resistance against parasitic plants.

ABA represents a critical phytohormone regulating plant resistance under biotic and abiotic stresses, coordinating responses by interacting with JA and SA signaling pathways [128, 129, 130]. *P. ramosa* infection increases ABA levels in both leaves and roots of infected tomato plants, with ABA‐glucose ester (ABA‐GE) levels also increasing significantly [129]. Despite elevated ABA, SL‐deficient lines show unaltered stomatal responses under infection, while wild‐type tomatoes exhibit increased stomatal opening and water loss, suggesting SLs may modulate ABA metabolism to enhance host defense [129]. ABA responses also occur during *O. crenata* parasitism of pea (*Pisum sativum*). Proteomic analysis of infected pea revealed significant accumulation of ABA‐responsive proteins, highlighting the role of ABA signaling in post‐attachment resistance [130].

Ethylene is a critical regulator of plant defense against biotic stresses, modulating both localized and systemic immune responses, which also plays a critical role in defending against parasitic plant infection [131, 132, 133, 134, 135, 136, 137, 138]. Parasitism by *C. reflexa* triggers ethylene emission in host plants, with CuRe1‐dependent ethylene induction serving as a hallmark of successful defense activation [124, 125]. Ethylene typically interacts with other hormones to coordinate defense responses. In *O. ramosa*‐*Arabidopsis* interactions, genes involved in JA and ethylene biosynthesis and signaling pathway are markedly upregulated while SA‐dependent defense is absent [131]. These findings demonstrate that hormone signaling pathways work coordinately to orchestrate comprehensive defense responses against parasitic plant invasion.

#### 3.2.3 Cell Wall Modification

Lignin is a complex polymer that strengthens plant cell walls and serves as a physical barrier against pathogen invasion [139, 140, 141, 142]. The function of lignin deposition in plant‐parasite interactions has been extensively studied. Prior research has demonstrated that resistant cultivars of *Vicia* spp. and faba bean can prevent *O. crenata* infection. These resistant plants fortify their endodermal cell walls through lignification, creating a physical barrier against parasitic invasion [143, 144].

A comparative metabolome analysis of *S. hermonthica*‐resistant rice (cultivar Nipponbare) and susceptible cultivar Koshihikari found significant accumulation of phenylpropanoid metabolites—particularly lignin monomers—in resistant plants [145]. Functional validation via RNAi‐mediated knockdown or overexpression of key lignin biosynthesis genes disrupted lignin composition and markedly reduced Nipponbare's resistance to *S. hermonthica* [145]. Genetic analyses revealed that lignin biosynthetic enzyme expression is also induced in *S. gesnerioides*‐resistant cowpea cultivars following parasitism [143]. Similarly, resistant sunflower cultivars respond to *O. cumana* infection by upregulating lignin key biosynthesis genes, including *F5H*, and genes coding peroxidases and cinnamyl alcohol dehydrogenase (CAD) [146]. These findings conclusively establish that enhanced lignin deposition and structural integrity at infection sites represent critical mechanisms underlying post‐attachment resistance against orobanchaceous parasitism.

In interactions between the stem parasitic plant dodder and its host, corresponding defense mechanisms have been identified. Studies of the post‐inoculation resistance mechanism using the tomato resistant variety Heinz as a model revealed that resistant tomato cultivars trigger localized lignification in the stem cortex at dodder attachment sites, forming a physical barrier to block haustorium invasion into host tissues [121]. The study identified key factors associated with lignin accumulation, including *LIGNIN INDUCTION FACTOR 1* (*LIF1*), *CUSCUTA RESISTANCE LRR RECEPTOR‐LIKE KINASE 1* (*CuRLR1*), and *SlMYB55*. Meanwhile, *SlWRKY16* was found to be upregulated following dodder infection, potentially suppressing *LIF1* function to negatively regulate resistance, establishing a dynamic homeostasis mechanism [121]. Through in‐depth analysis of these critical components, the study ultimately elucidated that tomatoes employ a four‐factor regulatory module (LIF1/SlMYB55/CuRLR1/SlWRKY16) to coordinate lignification‐based resistance, precisely interrupting dodder parasitism through a molecular mechanism [121].

Beyond lignin fortification, host defensive responses encompass diverse cell wall modifications, including structural protein cross‐bridging, callose accumulation, and suberization processes [146, 147, 148]. Studies demonstrated that resistant pea genotypes enhance H_2_O_2_ production and peroxidase enzymatic activity to facilitate oxidative cross‐bridging of structural proteins [146]. This protein cross‐bridging within cortical cell layers creates physical barriers that prevent *O. crenata* tissue penetration [146]. Similarly, callose accumulation was observed in resistant broad bean and pea plants, strengthening cellular boundaries to block *O. crenata* invasion [144, 146]. Comparable cross‐bridged cell wall proteins were identified in resistant sunflower genotypes as a mechanism to counter *O. cumana* parasitism [112]. Additionally, these resistant sunflower lines exhibit cell wall thickening at parasitic contact zones through suberin incorporation, creating impermeable barriers that prevent haustorial penetration and restrict parasitic access to endodermal tissues [112]. These findings indicate that resistant host plants typically deploy combinations of cell wall‐strengthening strategies to establish layered defense mechanisms, ensuring comprehensive protection against parasitic plants.

#### 3.2.4 Non‐Host Resistance

Beyond the specific pre‐attachment and post‐attachment resistance strategies discussed above, some plant species exhibit nonhost resistance (NHR), showing complete immunity to certain parasitic species regardless of infection pressure [149, 150, 151]. Notable examples of incomplete parasitic establishment include interactions between *S. hermonthica* and *Arabidopsis*, and between *O. minor* and *Lotus japonicus*, where initial invasion attempts fail to result in successful parasitism, which likely represents NHR [152]. Most eudicots possess immunity against multiple *Striga* species, such as *S. hermonthica* and *S. asiatica*, with the exception of *S. gesnerioides*—a unique parasite capable of infecting legumes, Convolvulaceous plants, and other eudicot families [153]. Parallel observations occur in interactions between *O. minor* and *L. japonicus*, where the parasite penetrates root tissues but fails to form storage organs owing to disrupted vascular connectivity [154]. The underlying causes remain debated: whether host plants actively disrupt parasitic connections or parasites lack essential factors for vascular integration.

In contrast to specialized parasites, generalist species like *P. japonicum* exhibit broader host ranges, successfully colonizing both monocots and eudicots [155]. However, their invasion of *L. japonicus* is thwarted by lignin‐based barriers at interaction sites. Although the molecular basis of nonhost resistance remains elusive [154], deciphering these mechanisms could revolutionize the development of parasite‐resistant crops, offering durable solutions to combat destructive plant parasites.

## 4 Conclusion and Future Perspectives

The intricate molecular dialogues between parasitic plants and their hosts, spanning diverse taxonomic groups from the root parasites of Orobanchaceae to the stem parasites of Convolvulaceae, underscore the evolutionary arms race that has shaped plant‐plant interactions. Over the past decade, significant strides have been made in deciphering mechanisms underlying parasitic plant germination, host detection, haustorium formation, and resource translocation, as well as host resistance strategies, including molecular recognition systems, hormone‐mediated defense responses, and cell wall modifications. These advances have deepened our understanding of parasitism and host defense across multiple parasitic plant lineages, both illuminating the co‐evolutionary dynamics between parasites and hosts and identifying actionable targets for mitigating agricultural losses caused by these devastating plant parasites.

Environmental factors, particularly soil nutrient status, have emerged as critical drivers of parasitic plant outbreaks through recently elucidated molecular mechanisms. Phosphorus deficiency has been identified as the primary environmental trigger for parasitic plant infestations, operating through upregulation of SL biosynthetic pathways that cause crop roots to exude increased levels of germination stimulants [156]. Recent groundbreaking work revealed that phosphorus‐deficient conditions induce expression of root specific ABC transporters responsible for SL secretion [98]. Strategic phosphorus application can directly reduce SL exudation, suppressing parasite germination at its source. Moreover, rising temperatures could expand the geographic range of thermophilic parasitic species, while changing rainfall patterns may affect soil moisture conditions critical for parasitic plant germination. Thus, climate change introduces additional complexity to parasitic plant management [157].

In addition to soil nutrient status, the soil microbiome has been revealed to function as an indirect layer of the plant immune system against parasitic plants, offering sustainable biological control opportunities. Natural soil microbiomes significantly suppress *S. hermonthica* infection compared to sterilized soils [158]. Rhizosphere microbiomes suppress *Striga* through multiple coordinated pathways, including enhanced endodermal suberization, microbial degradation of haustorium‐inducing factors, direct antibiosis, and competition for nutrients and microsites. These findings suggest that microbiome‐based strategies could be optimized through targeted microbial inoculation or soil amendment approaches.

While current research predominantly treats post‐attachment host defense against parasitism through the lens of pathogen infection models, fundamental differences exist between pathogen invasion and parasitic plant interactions. Unlike bacterial or fungal pathogens, parasitic plants are phylogenetically close to their hosts, creating challenges in distinguishing host‐derived signals from parasite‐derived signals. Pattern recognition receptors (PRRs) must target more specific, less conserved molecules, exemplified by the tomato CuRe1 receptor's specific recognition of Crip21 peptide from *Cuscuta* cell wall proteins [124, 125]. Unlike typical pathogen effectors, parasitic plant effectors like SHR4z from *S. gesnerioides* employ sophisticated molecular mimicry to bind and degrade host proteins such as VuPOB1, suppressing immunity [118]. At its core, parasitic plant interactions are defined by their bidirectionality: they form permanent vascular connections, which enable the sustained molecular exchange of mRNAs, proteins, and metabolites [82, 83, 84, 159]. This unique feature creates an interaction paradigm that is fundamentally distinct and requires distinct defense strategies.

Achieving long‐term, sustainable control of parasitic plants from both the Orobanchaceae and Convolvulaceae families may require integrated management strategies that synergize genetic, chemical, and ecological interventions. Genetic approaches utilize gene editing and transgenic technologies to engineer crop resistance at both pre‐attachment stages and post‐attachment stages. Chemical interventions deploy synthetic compounds to trigger suicidal germination—depleting soil seed banks—or selectively disrupt haustorium development. Ecological strategies manipulate soil nutrient status, optimize agronomic practices, and harness beneficial microbiomes to suppress parasitism indirectly. Together, these complementary approaches offer a multi‐layered framework adaptable to diverse cropping systems and regional constraints.

At the pre‐attachment stage, SLs manipulation represents the primary genetic intervention through three mechanisms: reducing SL synthesis, altering SL composition, and blocking SL secretion. While synthesis reduction causes developmental defects, the latter two strategies face distinct limitations. Altering SL composition lacks field validation, particularly in maize under African conditions, and raises concerns about inadvertently triggering germination of non‐target parasitic species. Blocking SL secretion remains untested against highly aggressive *S. hermonthica* races, and whether this strategy could compromise arbuscular mycorrhizal (AM) symbiosis that critical for crop growth in nutrient‐poor, arid environments needs further verification.

Strategic improvements could address these constraints through targeted interventions. First, co‐disrupting the SL signaling repressor D53 alongside biosynthetic genes might preserve normal development despite reduced SL synthesis [160]. Second, multi‐location field trials across Africa combined with precision engineering toward low‐activity SL variants could validate compositional approaches while minimizing risks of non‐target parasite germination. Third, manipulating substrate‐specific transporters by selectively blocking parasite‐stimulating SLs while maintaining AM‐promoting SLs could balance crop productivity and parasite resistance. This requires verifying two key points: whether SL transporters display substrate specificity, and whether parasites and AM fungi recognize distinct SL structural classes. At the post‐attachment stage, a breakthrough achievement in cross‐family transfer of NLR immune receptors offers transformative potential. Functional sensor‐helper NLR pairs now transfer successfully across angiosperm families, enabling deployment of characterized resistance genes like *VuRSG3‐301* into diverse crops, while synthetic “Pikobodies”—representing another major innovation—create designer immunity against parasitic effectors or haustorium signals [161, 162].

Chemical interventions provide flexible deployment options but face critical implementation barriers. Production costs remain prohibitively high, with most synthetic compounds confined to laboratory stages and inaccessible to smallholder farmers facing the greatest parasitic burden. The narrow chemical repertoire, which focuses predominantly on suicidal germination inducers exhibits uncertain field efficacy and requires potentially high application rates that further drive‐up expenses. Species‐specific activity exacerbates this challenge: agents effective against one parasitic lineage often prove ineffective against others, which in turn increases development costs and restricts practical applicability. Emerging technologies and ecological insights provide promising avenues for addressing these challenges. Synthetic biology approaches that optimize biosynthetic pathways for microbial production could significantly reduce costs. Expanding beyond germination stimulants to include SL receptor antagonists and haustorium inhibitors would diversify control mechanisms. Most critically, targeting conserved haustorium development pathways or SL perception mechanisms shared across parasitic lineages, instead of species‐specific targets, would achieve broad‐spectrum, durable efficacy with enhanced cost‐effectiveness.

Ecological strategies require moving beyond isolated interventions to integrated agronomic systems. While phosphorus management and microbiome manipulation offer targeted solutions, their effectiveness depends on coordinated deployment with crop rotation, intercropping, and tillage practices that disrupt parasitic life cycles. Trap cropping represents another promising ecological approach: deploying plants that secrete high‐activity SLs yet resist parasitic attachment (trap crops such as cowpea and cotton), or tolerant hosts that sustain parasitism without significant yield loss (catch crops), stimulates parasitic seed germination and attachment while ultimately suppressing parasite reproduction through seed bank depletion [163, 164]. Field trials have demonstrated that rotation with appropriately selected trap crop cultivars—such as cowpea variety TVX 1850‐01F against *S. hermonthica*—can reduce subsequent parasitic infestation to nearly undetectable levels within a single season [163]. Biocontrol strategies using antagonistic soil bacteria or fungi with selective antiparasitic activity provide sustainable alternatives. Critically, these approaches must balance efficacy with farmer accessibility, as complex multi‐input systems risk low adoption among smallholders, the group most vulnerable to high parasitic pressure. However, ecological strategies face an urgent future challenge, climate adaptation. Despite well documented temperature‐dependent germination patterns, the molecular mechanisms underlying the link between temperature sensing and *Striga* dormancy release remain elusive. Identifying temperature‐responsive genetic pathways and their natural variation across parasitic populations would facilitate the development of predictive models under warming scenarios and guide breeding for climate‐resilient resistance. Without this mechanistic foundation, ecological strategies risk becoming obsolete as climatic conditions alter parasitic distribution ranges and phenologies into uncharted territory.

Sustainable management strategies must reconcile agricultural protection with the significant medicinal value of parasitic plants. *Cuscuta* species have been valued in Traditional Chinese Medicine for over 2000 years, with modern analyses revealing bioactive flavonoids, polysaccharides, and lignans exhibiting hepatoprotective and immunomodulatory effects [165, 166, 167, 168, 169]. Similarly, *Orobanche* species demonstrate anti‐inflammatory properties, with several incorporated into traditional pharmacopoeia. Intriguingly, their chemical profiles vary significantly depending on host plants due to haustorial absorption, presenting opportunities for optimizing bioactive compound production through controlled cultivation [170, 171, 172].

The convergence of genetic, chemical, and ecological innovations has fundamentally transformed parasitic plant management. Integrating targeted genetic interventions, diversified chemical strategies, and microbiome‐based ecological practices enables a shift from reactive mitigation toward proactive, sustainable control. This multi‐layered approach, which is adaptable to regional constraints and grounded in mechanistic insights, can safeguard global food security, while such insights can also facilitate the cultivation of high‐value medicinal parasitic plants. Future progress demands coupling molecular precision with field validation, crop protection with biodiversity conservation, and biological efficacy with economic accessibility.

## Author Contributions

F.Y. conceptualized the paper. J.S. and F.Y. drafted and revised the manuscript. Q.X. provided valuable suggestions.

## Conflicts of Interest

The authors declare no conflicts of interest.
